# Expression of resilience, coping and quality of life in people with cancer

**DOI:** 10.1371/journal.pone.0236572

**Published:** 2020-07-29

**Authors:** Patricia Macía, Mercedes Barranco, Susana Gorbeña, Ioseba Iraurgi

**Affiliations:** 1 Department of Personality, Evaluation and Psychological Treatments, University of Deusto, Bilbao, Biscay, Spain; 2 Spanish Association Against Cancer (Provincial Office of Biscay), Bilbao, Spain; University of Auckland, NEW ZEALAND

## Abstract

Considering the importance of coping strategies and resilience in adapting to the stress caused by cancer, the objective of this research is to explore which coping strategies are the most used, in order to know whether different groups of levels of resilience and an appropriate coping style are related to a higher quality of life and better adaptation to the disease. There were 74 participants with cancer in this study (79.7% of them were women) ranging in age from 29 to 85 years (*M* = 50.9). Different instruments were used to measure the resilience construct (ER-20 items Resilience Scale), coping strategies (Cognitive Emotion Regulation Questionnaire-Short) and quality of life (General Health Questionnaire). People with higher resilience showed higher scores in the use of adaptive strategies, being acceptance and positive revaluation the most frequent ones. Regarding perception of quality of life, people with lower resilience showed statistically significant differences in the dimensions of pain and general health, which were likewise the most common ones for people with lower resilience. A significant association has been demonstrated between resilience and an adaptive coping, which at the same time are positively linked to quality of life of people with cancer. This study provides information about how different groups of resilience levels are related with coping and quality of life in people with cancer. It could be useful information for psychologists in the oncological area who have to take decisions in the clinical context. A practical consequence would involve trying to modify the type of coping, as well as increasing the level of resilience in people with cancer, in order to achieve a better adjustment to the disease.

## Introduction

It has been documented that people suffering from cancer disease, in most cases, experience high level of stress that can provoke negative symptoms such as anxiety, depression or fear. [1,2] In fact, some people may live through it as a traumatic experience that threatens their physical and psychological well-being. [3] The impact that this will have on them will vary according to the resources that they use. In this respect, the function that different coping strategies have when adapting to this type of oncological processes has been investigated. [4–7] An appropriate adjustment to the disease process implies an effective control of the patient's behaviour and emotions, mental flexibility, as well as an appropriate interaction with the patient's environment. All this will favour the management of their own resources in order to cope with the stressful situation. [8–10] Lazarus and Folkman [6] define “coping” as the cognitive and/or behavioural efforts that are made to deal with stressful or difficult situations that test people’s resources. Conceptually they distinguish two types of coping: problem-oriented or adaptive coping (which pretends to manage or modify the problem that is causing distress, facing stressor in different ways, etc.) and emotion-oriented or disadaptive coping (which tries to regulate the emotional response to adversity). [11]

Literature has demonstrated that certain coping strategies are more adaptive and widely used than others, leading to a more constructive, positive and active coping processes. [11,12] In fact, coping effectiveness can vary depending on several factors, such as cancer stage, time since diagnosis, medical treatment, etc. For instance, some authors have found that coping strategies such as acceptance, positive reappraisal and seeking social support are associated with higher adaptation, well-being and quality of life in cancer. [13–15] On the contrary, disadaptive coping strategies such as self-blame, avoidance and negation are related to poorer mental health outcomes. [16,17]

Oliveros-Áriza, Barrera, Martínez and Pinto [18] evaluated coping strategies used by people diagnosed with different types of cancer, taking into account whether these coping processes were problem-oriented or emotion-oriented. Most individuals were placed in the “problem-oriented” category and they used strategies such as problem solving and seeking social support. Patients that could potentially recover from the disease used those strategies in order to adapt themselves, since this type of coping favoured their adherence to treatment and therefore, it increased expectations of an improvement. Nevertheless, they assume that “emotion-oriented” strategy is equally important and adaptive, since it helps to accept certain situations. This strategy allows individuals to keep hope and in cases where recovery is not possible, it helps individuals to adapt themselves to chronic pain. [19] These types of coping strategies can be more or less adaptive at any given time and depending on their use. [20] Nevertheless, it is also important to explore which type of strategies could be disadaptive, with the goal of avoiding risk of maladaptive health behaviours and greater emotional distress. [21]

Saita, Acquati and Kayser [22] investigated coping strategies in Italian breast cancer women considering the influence of personality traits and close relationships. They found that perceived strength of relationships predicted the use of active and adaptive coping strategies, being the women with high assertiveness and social anxiety who most used an active coping style. These results showed that patients adopted an active coping when experimenting high levels of distress (anxiety, for example) in different stressful situations. They highlighted an active coping pattern in patients who considered the disease as a challenge which threatens their assertiveness and social sphere.

The use of particular coping strategies in cancer disease, which can be life-threatening and leads individuals to make vital decisions about their own health, influences on patients´ perception about their illness. [21] Coping helps adapting to the disease process and is important in achieving or maintaining quality of life. However, some disadaptive coping strategies as rumination, self-blame and suppression have been linked to lower mental health (more depressive and anxiety symptoms) and poorer physical and psychological quality of life. [23,24,21]

Another concept closely linked to adjustment and coping with cancer is resilience. It has been defined as the ability to cope with traumatic and stressful events and to overcome them in an effective and positive way. [25,26] Resilient people are able to emerge even stronger from difficult situations, improving their coping strategies and adaptation to the adverse situation. [20] Resilience involves protective personal attributes including cognitive flexibility, positive emotions and an active coping. [27] In this regard, Smith, Saklofske, Keefer and Tremblay [28] found out that individual differences in resilience moderated the effects that the fact of actively coping with the disease has on the negative affect or on depression, whereas the fact of coping with the disease in an emotion-oriented way was not related to resilience. In fact, emotion-oriented coping strategies were linked to poorer psychological outcomes.

In this context, Ye et al [29] studied the mediating effect of resilience in the association between emotional distress and quality of life in the case of patients with cancer. They found out that resilience was a predictor of high levels of quality of life in patients. Likewise, results showed that high levels of resilience were linked to a 64% reduction in the risk of emotional distress, producing a significant buffering effect of resilience on depression. When a patient has some personal characteristics that imply being resilient, these could help him/her to better tolerate negative feelings and emotions. [30] This tolerance, in turn, would promote a way of coping better with cancer disease. [31]

To our knowledge, this particular concept of resilience has not been fully investigated taking into account differences by resilience groups in relation to coping and quality of life in cancer patients. Our aim is to explore the expression -as well as the connection- of different levels of resilience in people with cancer and the way they cope with the disease, based on the following hypothesis: a greater resilience will be associated with an adaptive coping during the disease process. We also intend to explore the way in which this would be related to a better adaptation to the disease in terms of quality of life. This information could be important in the clinical practice, since considering personal differences in cancer patients would facilitate a more adapted and personalized prescription of psychological care. Treatments would be more adjusted to the specific needs of each patient, contributing to a better adaptation to disease and, consequently, an increase and improvement in their quality of life.

## Materials and methods

### Ethics

This study was carried out in accordance with the recommendations of the Helsinki declaration and the guidelines of the International Committee of Medical Journal Editors. All subjects gave written informed consent in accordance with the Declaration of Helsinki. The protocol was approved by the University of Deusto Research Ethics Committee.

### Participants

The participants in the study are 74 people (79.7% of them are women) who have been diagnosed with cancer [mainly breast cancer (51.0%), lung (26.6%), colon (14.3%) and other (8.1%)], ranging in age from 29 to 85 years (M = 50.9). Most of them are married (68.9%) and have a university degree (58.1%) (Table 1). All of them were users of the supporting and/or counselling services provided by the Spanish Association Against Cancer (AECC) in Biscay and in the 74.3% of cases they were receiving active oncological treatment.

**Table 1:** Means (M) or percentages, Standard Deviations (SD) and sociodemographic variables ranges for the sample of people with cancer and by resilience groups.

Variable		Total		Low	Medium	High
		(n = 74)		(n = 22)	(n = 27)	(n = 25)
		M/n	SD/%	M (or %) ± SD		
Age		50.9	9.7	54.86 ± 10.3	50.26 ± 9.8	48.10* ± 8.2
Gender (%)	Woman	59	79.7	86.4	66.7	88.0
	Man	15	20.3	13.6	33.3	12.0
Studies (%)	Primary school	11	14.8	20.0	11.5	12.0
	Secondary school	5	6.7	5.0	0.0	16.0
	Bachelor	4	5.4	15.0	3.8	0.0
	Professional training	11	14.8	10.0	15.4	20.0
	University	43	58.3	50.0	69.3	52.0
Civil status (%)	Single	11	14.8	20.0	23.1	4.0
	Married, in couple	51	68.9	55.0	65.4	84.0
	Separated, divorced	5	6.8	15.0	0.0	8.0
	Widower	7	9.5	10.0	11.5	4.0
Treatment (%)	Without Treatment	19	25.7	27.3	29.6	20.0
	Tt in the last 6 months	55	74.3	72.7	70.4	80.0

n = sample size, M = mean, SD = standard deviation.

*p<0.05.

### Procedure

Participants were found through AECC. The association asked them to participate in the study by replying to a questionnaire. The association informed people about this study and its goals through its scheduled contacts or by email, and invited them to participate on a voluntary basis. Legal holders and technical teams of AECC had given their approval to the study and participants had given informed consent.

Participants replied to a questionnaire (see Instruments) and on average it took them 30 minutes. The questionnaire could be completed in paper, at the premises of the association or on-line, if it suited them better.

### Instruments

The information was collected using the questionnaire technique, which included socio-demographic data, characteristics of the oncological disease and three psychometric instruments to investigate the variables they were interested in: coping, resilience and quality of life in people with oncological disease.

#### Resilience

In order to evaluate resilience, a scale with 20 items (ER-20 items Resilience Scale) was used. [32] This scale inquired about four different patterns of the construct of resilience: the dispositional pattern, the relational pattern, the situational pattern and the philosophical pattern. [25] The dispositional pattern makes reference to physical factors such as intelligence, health and temper. The relational pattern makes reference to the characteristics of relationships and roles that have an influence on resilience, such as developing supportive relationships or personal privacy. The situational pattern reflects the way the participant approaches stressful situations, which is manifested through his/her abilities for cognitive assessment, for problem solving, etc. Finally, the philosophical pattern is manifested by personal beliefs, including self-knowledge and self-reflection. Answers to the statements were provided using a Likert scale that included five answer options, depending on the frequency and/or intensity with which they are manifested (from 0 –*Never or hardly ever* to 4 –*Quite often or often*). This instrument allows the establishment of a complete indicator of resilience, in a decimal scale. Therefore, a higher score indicates a greater expression of this ability. An Exploratory Factorial Analysis (EFA) showed a high participation of items to a common factor, demonstrating the unidimensionality of the scale. The analysis of the correlation matrix showed a KMO coefficient (Kaiser-Meyer-Olkin) of .81; and Bartlett's sphericity test [*χ*^2^_(190)_ = 723.9; *p*≤ .001] was significant, indicating the suitability of the matrix to be factored. Likewise, the MAP indices and the Parallel analysis have indicated the extraction of a single factor. A Confirmatory Factorial Analysis [33,34] (CFA) for a one single factor model presents results that suggest that the adjustment indices obtained have been adequate: *χ*^2^_(170)_ of Satorra-Bentler = 266.22; *p* < .001, with a normalized adjustment index (1.56), CFI = .90, GFI = .92, RMSEA = .088 (.067 to .107). The internal consistency achieved in this study was a Cronbach´s alpha value of .87. [35]

#### Coping strategies

The Cognitive Emotion Regulation Questionnaire scale [36] (CERQ- Cognitive Emotion Regulation Questionnaire-Short) was used in order to evaluate coping strategies. This scale enables the evaluation of the cognitive assessment of the subject when facing threatening or stressful life situations. The CERQ has shown good psychometric properties, with Cronbach´s alpha coefficients over .80. Besides, it has proved a good factorial, construct and discriminative validity. [36] Answer categories for each of the items range from 1 [hardly ever or never] to 5 [(almost) always]. For this study, the shorter version of 18 items was used. The original scale has 36 items that are divided into 9 subscales and they conceptually bring together two types of coping strategies: adaptive coping and disadaptive coping. This version was adapted into Spanish [37] and it has the same dimensional structure: the adaptive strategies (*α* = .80) [acceptance (*α* = .91), focusing on the planning (*α* = .63), positive refocusing (*α* = .89), positive revaluation (*α* = .70), putting the situation into perspective (*α* = .87)] and/or the disadaptive ones (*α* = .67) [self-blame (*α* = .73), blaming others (*α* = .81), rumination (*α* = .75) and catastrophism (*α* = .93)]. The internal consistency obtained in this study -for all the dimensions- showed Cronbach´s alpha value of .70.

#### Quality of life

For the evaluation of the subjective quality of life, the General Health Questionnaire SF-12 was used. It was designed by Ware, Kosinski and Keller [38], based on the SF-36 [39], and it was adapted into Spanish by Alonso, Prieto and Antó. [40] It evaluates eight dimensions of health: Physical Functioning (PF), Role limitations due to Physical health problems (RP), Social Functioning (SF), Bodily Pain (BP), Mental Health (MH), Role limitations due to personal problems or Emotional distress (RE), Vitality (VI) and General Health (GH). The overall score is calculated by summing the scores obtained with the Likert scale, measured in ascending order. Consequently, a higher score indicates a higher perception of quality of life. The scale shows a profile of health status that is based on the score obtained in each one of the eight dimensions that are analysed in a centesimal scale. Additionally, the instrument permits the generation of two total scores (physical health and mental health–TPC (*α* = .58) and TMC (*α* = .82), respectively) which are expressed in standardised T scores. These scores were obtained using specific algorithms. In this case, the algorithms that were used were provided by the group of people that adapted the instrument in Spain, under the direction of the Municipal Institute of Medical Research of Barcelona. The internal consistency obtained in this study showed a Cronbach´s alpha value of .77.

### Statistical analyses

Every questionnaire was virtually completed in its entirety but in some cases (12.1%) the answer to some items was omitted. In order not to miss any case, the median value of the answers that were given to the indicator's items was applied to lost values, provided that missing values were not higher than the 10% of the items that are included in such indicator. [41,42]

On the basis of the indicator of resilience, which is shown in a decimal scale, a second indicator was created. This second indicator would allow creating three ordinal levels of resilience (low, medium and high). For this purpose, the tertiles of the distribution were taken into account and 22, 27 and 25 participants were grouped, respectively.

The mean value (M) and the standard deviation (SD) were used for the description of the scale variables, and the frequency (n) and percentage (%) were used for the description of nominal variables. The chi-square test was used for comparing proportions according to resilience levels, while the robust Brown-Forsythe analysis of variance was used for comparing the mean values. Student's *t* test was used for comparing the average of both groups and timely corrections were made in the event of heteroscedasticity. Besides, Cohen *d* was calculated in order to know the effect size of the differences between groups. Finally, the degree of association between variables was established by calculating the Pearson correlation coefficient in the case of scale variables, and by calculating the point-biserial coefficient when one of the variables was dichotomous. SPSS software version 22 was used to perform these statistical analyses. [43]

## Results

### Sociodemographic variables ranges and by resilience groups

For a possible range of resilience values from 0 to 10, the average value detected among participants was 5.87 (SD = 1.5). Taking the classification by resilience levels into account, the first tertile (low level of resilience) would involve scores between a minimum of 2.50 and 5.15, the medium level would involve scores between 5.29 and 6.32 and the high level would involve scores between 6.47 and a maximum of 9.56. The socio-demographic variables did not show any difference when they were contrasted with resilience levels (Table 1), except in the case of age [*F*_(2)_ = 3.07; *p* = 0.053]. In that case, it was noted that people with a low resilience level (M = 54.8) were older than people with a higher resilience level (M = 48.1). Although it has not been statistically significant, a slightly higher proportion of women has been observed among participants with a low (86.4%) and high (88%) resilience level (*χ*^*2*^ = 32.54; *p* = 0.44). In addition, a higher proportion of participants with a high resilience level (80%) has been observed among those who were receiving active treatment (*χ*^*2*^ = 33.50; *p* = 0.394).

### Correlations and student t-test for resilience with coping strategies and quality of life

Table 2 shows that practically all the adaptive coping strategies were positively linked to resilience (*r* = 0.63; *p*<0.001). A greater connection to strategies of positive refocusing (*r* = 0.59; *p*<0.001) and positive revaluation (r = 0.45; *p*<0.001) was also observed. Concerning the totality of disadaptive strategies, self-blame was the only strategy that was linked to resilience in a statistically significant way (*r* = -0.32; *p* = 0.006), even though the totality of disadaptive strategies showed a negative relation to resilience.

**Table 2:** Correlations and student *t-*test for resilience factor with coping strategies and quality of life.

Variable	M ± SD	Resilience	*r*		*t (d)*	
			Age	Studies	Actual Treatm.	Gender
					(0 = No, 1 = Yes)	(1 = woman, 2 = man)
**Resilience**	5.87 ±1.50	1.00*	-0.25*	-0.13	-0.60 (0.17)	0.66 (0.21)
**Adaptive Coping Strategies**	2.30 ± 0.76	0.63*	-0.30**	0.02	0.18 (0.05)	1.28 (0.38)
Acceptance	2.76 ± 1.29	0.36*	-0.12	-0.11	0.31 (0.07)	1.11 (0.29)
Positive refocusing	2.05 ± 1.20	0.59*	-0.23*	-0.12	0.24 (0.09)	1.76 (0.54)
Planning	2.20 ± 1.08	0.22	-0.17	0.05	0.81 (0.21)	0.12 (0.03)
Positive Revaluation	2.59 ± 1.10	0.45*	-0.25*	0.12	0.93 (0.26)	1.55 (0.45)
Putting into perspective	1.93 ± 1.32	0.38*	-0.21	0.15	-1.44 (0.39)	-0.35 (0.10)
**Disadaptive Coping Strategies**	1.05 ± 0.60	-0.24*	0.10	-0.01	1.04 (0.26)	0.52 (0.15)
Self-blame	0.80 ± 0.88	-0.32*	0.08	0.08	-0.23 (0.07)	0.84 (0.26)
Rumination	1.93 ± 1.19	-0.11	0.06	0.08	0.95* (0.26)	0.24 (0.07)
Catastrophism	1.22 ± 1.25	-0.60	0.12	-0.15	0.80 (0.21)	-0.38 (0.10)
Blaming others	0.23 ± 0.57	-0.19	-0.07	0.03	0.76 (0.18)	1.96* (0.42)
**Quality of Life**						
PF-Physical Functioning	51.01 ± 31.09	-0.21	-0.16	0.43*	2.02 (0.54)	-0.32 (0.09)
RP-Physical Role Limitations	40.27 ± 21.52	-0.04	-0.21	0.28*	1.82 (0.46)	-0.62 (0.18)
BP-Body pain	66.22 ± 33.22	-0.30*	-0.13	0.47*	1.34 (0.36)	-1.60 (0.50)
GH-General Health	66.82 ± 21.70	-0.13	0.26*	-0.17	-0.30 (0.09)	-0.23 (0.07)
VI-Vitality	55.41 ± 19.74	0.10	-0.08	0.16	0.91 (0.23)	-0.10* (0.03)
SF-Social Functioning	63.85 ± 28.11	0.07	-0.18	0.18	0.82 (0.22)	-0.77 (0.21)
RE-Emotional Role Limitations	51.76 ± 21.03	-0.01	-0.17	0.32*	1.49 (0.38)	0.09 (0.03)
MH-Mental Health	55.81 ± 15.17	0.14	-0.08	0.15	-0.31* (0.09)	-0.06 (0.02)
TPC-Total Physical Component	42.63 ± 8.80	-0.34*	-0.09	0.41*	2.05 (0.56)	-1.18 (0.34)
TMC-Total Mental Component	40.42 ± 9.46	0.23*	-0.11	0.05	-0.09 (0.02)	0.18 (0.05)

M = mean, SD = standard deviation, *r* = Pearson correlation coefficient, *t* = *t*-Student.

*p<0.05.

On the other hand, it has been noted that age was negatively related to resilience (*r* = -0.25; *p* = 0.029) and to adaptive coping strategies (*r* = -0.30; *p* = 0.009). Besides, statistically significant differences were found regarding gender and the blaming strategy (*t* = 1.96; *p* = 0.02). The difference was bigger in the case of women. Table 2 also shows the results for the variable of quality of life in connection with resilience, as well as socio-demographic variables. Resilience was significantly and negatively associated with the perception of pain (*r* = -0.30; *p*<0.009), and in general, it was also negatively associated with the physical component of quality of life (*r* = -0.34; *p*<0.003). However, resilience was significantly and positively associated with the mental component (*r* = 0.23; *p*<0.047). Regarding age, it was noted that the older the participants were, the better their perception of health was (*r* = 0.26; *p* = 0.023).

### Comparison of means of coping strategies by resilience tertiles

**Fig 1:**
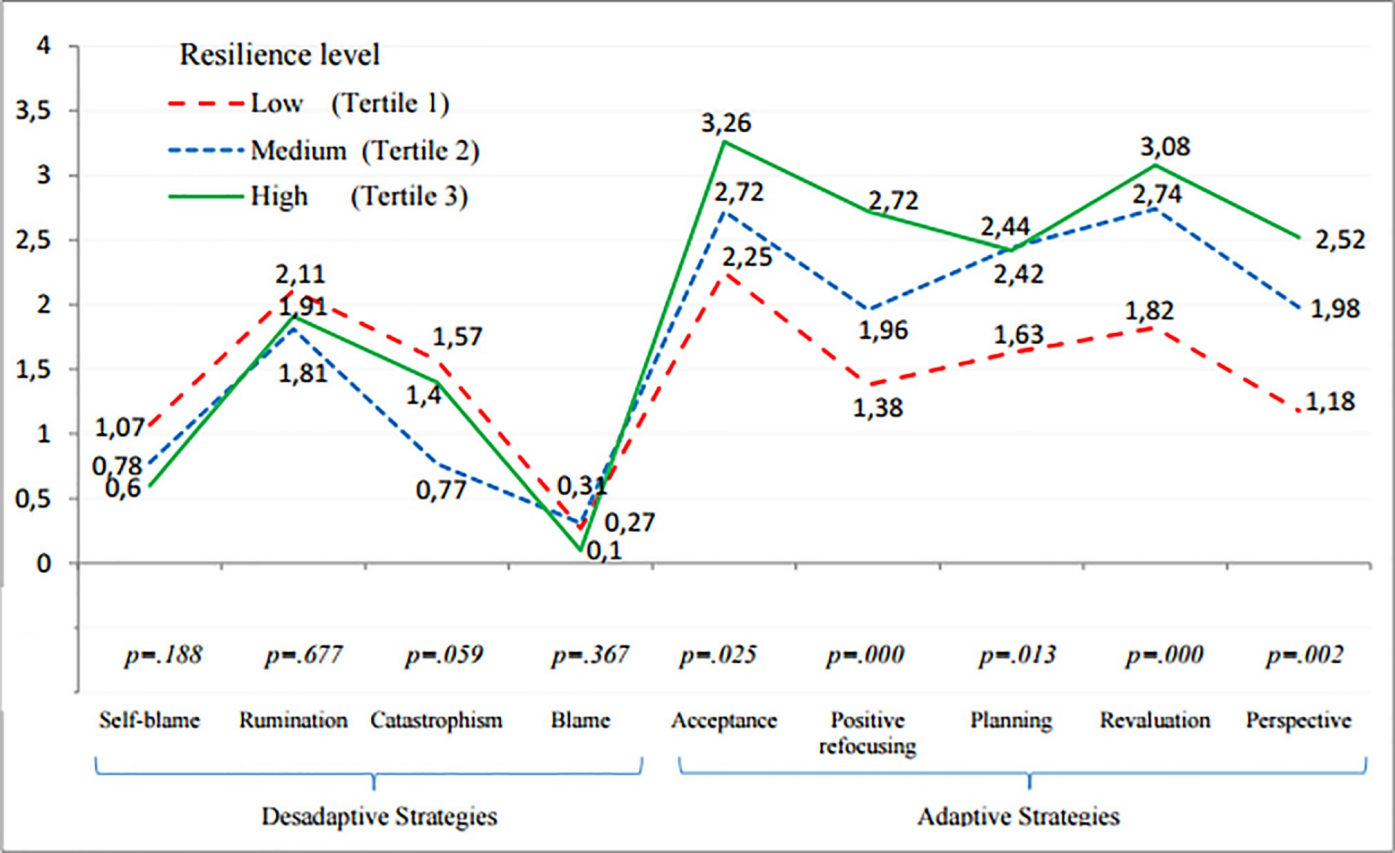
Comparison of means of adaptive and disadaptive coping strategies by resilience tertiles.

Fig 1 shows the differences between different resilience groups when using coping strategies. The five dimensions of adaptive coping strategies showed statistically positive significant differences between resilience groups, and the global index of adaptive coping resulted from the integration of all of them was likewise statistically significant [*F*_(2)_ = 20.41; *p*<0.001]. Effect sizes were strong (*d* = 1.19; *p*<0.001) for low and medium levels of resilience; for medium and high levels of resilience (*d* = 1.96; *p*<0.001); and for low and high levels of resilience (*d* = 0.70; *p* = 0.017) (Table 3). On the contrary, disadaptive strategies did not show statistically significant differences between the different groups. However, it was found a significant effect size between people with low and medium level of resilience when using this type of strategies (*d* = 0.65; *p* = 0.031), and specially, when using the strategy of catastrophism (*d* = 0.71; *p* = 0.02).

**Table 3:** Effect sizes for resilience groups with coping strategies and quality of life.

	Q_1_ (n = 22)		Q_2_ (n = 27)		Q_3_ (n = 25)		ANOVA	Post-hoc			
	M	SD	M	SD	M	SD	*F*	*df*	Q_1-_ Q_2_	Q_1-_ Q_3_	Q_2-_ Q_3_
									*d*	*d*	*d*
**Adaptive Coping Strategies**	1.65	0.59	2.37	0.64	2.80	0.61	20.41*	2	1.19*	1.96*	0.70*
Acceptance	2.25	1.32	2.72	1.36	3.26	1.01	3.89*	2	0.36	0.89*	0.46
Positive refocusing	1.39	0.96	1.96	1.21	2.72	1.05	8.93*	2	0.53	1.35*	0.68*
Planning	1.63	0.82	2.44	1.19	2.42	1.01	4.64*	2	0.79*	0.87*	0.02
Positive Revaluation	1.81	1.25	2.76	0.92	3.08	0.73	10.41*	2	0.90*	1.29*	0.39
Putting into perspective	1.18	1.22	1.98	1.27	2.52	1.18	7.01*	2	0.65*	1.14*	0.45
**Disadaptive Coping Strategies**	1.26	0.55	0.92	0.52	1.00	0.69	2.04	2	0.65*	0.42	0.13
Self-blame	1.07	0.81	0.78	0.87	0.60	0.92	1.71	2	0.35	0.55	0.21
Rumination	2.11	1.35	1.81	0.98	1.90	1.26	0.39	2	0.26	0.16	0.08
Catastrophism	1.57	1.35	0.78	0.94	1.40	1.36	2.94	2	0.71*	0.13	0.54
Blaming others	0.27	0.53	0.31	0.76	0.10	0.29	1.02	2	0.06	0.41	0.37
**Quality of Life**											
PF-Physical Functioning	53.41	33.89	54.63	24.06	45.00	35.35	0.71	2	0.04	0.25	0.33
RP-Physical Role Limitations	41.36	21.22	38.89	24.07	40.80	19.56	0.09	2	0.11	0.03	0.08
BP-Body pain	73.86	28.32	72.22	28.87	53.00	38.41	3.18*	2	0.06	0.63*	0.58*
GH-General Health	76.14	14.39	59.81	25.32	66.20	20.53	3.70*	2	0.79*	0.57	0.28
VI-Vitality	54.54	22.41	54.07	20.62	57.60	16.65	0.23	2	0.02	0.16	0.19
SF-Social Functioning	57.95	23.64	67.59	25.77	65.00	33.85	0.74	2	0.40	0.24	0.09
RE-Emotional Role Limitations	51.36	24.55	51.48	19.94	52.40	19.64	0.02	2	0.01	0.05	0.05
MH-Mental Health	54.09	17.90	55.56	15.27	57.60	12.67	0.31	2	0.09	0.23	0.15
TPC-Total Physical Component	45.40	7.90	42.98	7.16	39.80	10.49	2.51	2	0.33	0.61*	0.36
TMC-Total Mental Component	38.17	11.36	40.27	9.03	42.54	7.84	1.27	2	0.21	0.46	0.27

M = mean, SD = standard deviation, *F* = *F* de Fisher-Snedecor, d*f* = degrees of freedom, *d* = Cohen´s *d*, *Q*_*1* =_ low level of resilience, *Q*_*2*_ = medium level of resilience, *Q*_*3*_ = high level of resilience.

*p<0.05.

### Comparison of means of quality of life by resilience tertiles

**Fig 2:**
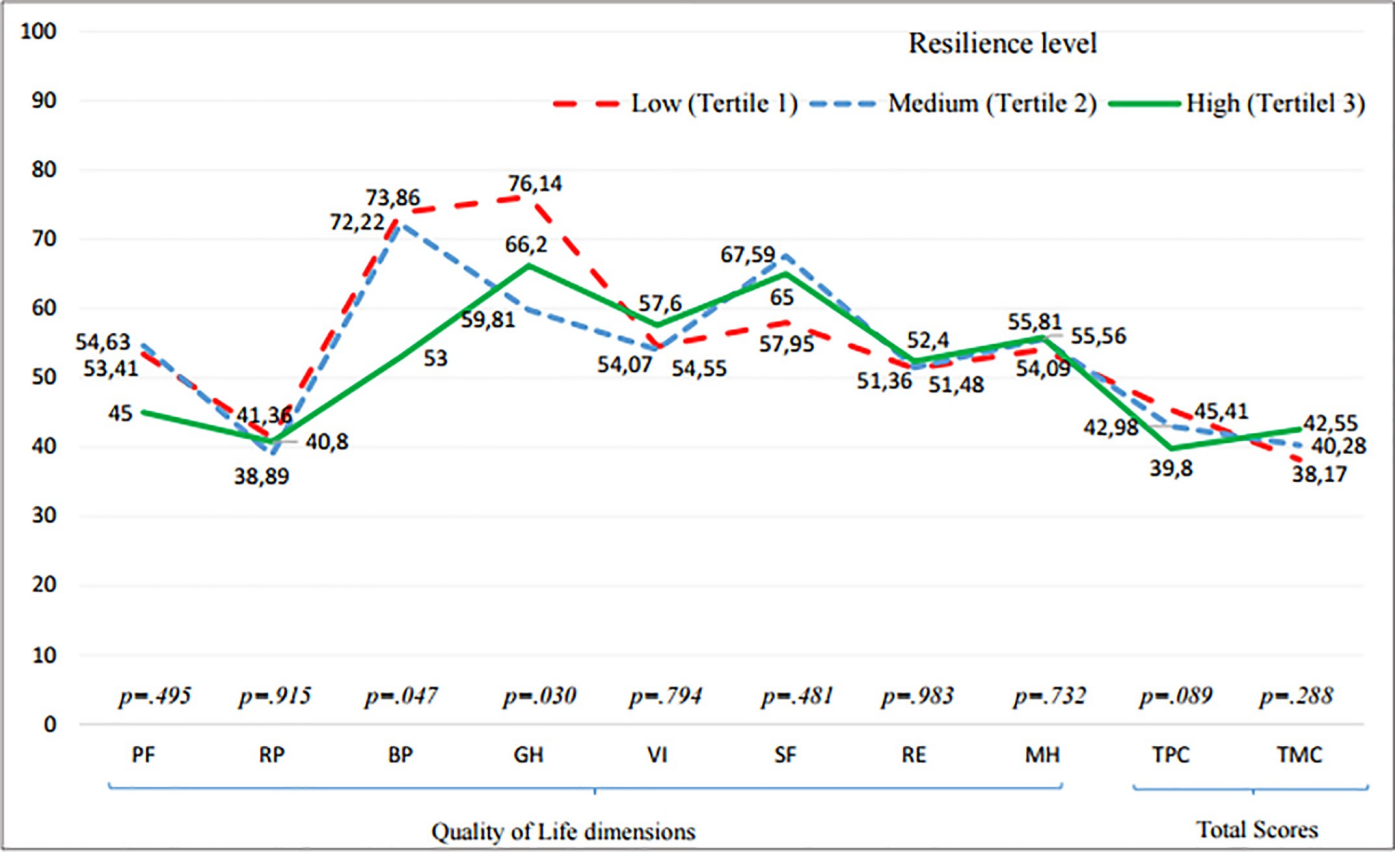
Comparison of means of quality of life by resilience tertiles. PF-Physical Functioning, RP-Physical Role Limitations, BP-Body pain, GH-General Health, VI-Vitality, SF-Social Functioning, RE-Emotional Role Limitations, MH-Mental Health, TPC-Total Physical component, TMC-Total Mental Component.

On the other hand, Fig 2 shows the differences in the perception of quality of life for the three resilience groups. The dimensions of bodily pain [*F*_(2)_ = 3.18; *p* = 0.047] and general health [*F*_(2)_ = 3.70; *p* = 0.03] were the only ones that showed statistically significant differences. The dimension of pain showed significant effect sizes only for the comparison of low and high level of resilience (*d* = 0.63; *p* = 0.041), and medium and high level of resilience (*d* = 0.58; *p* = 0.046). While a significant effect size was only observed between low and medium level of resilience in general health (*d* = 0.79; *p* = 0.009) (Table 3). Besides, it must be also remarkable that although the total physical component of quality of life does not show statistical differences [*F*_(2)_ = 2.51; *p* = 0.089], a statistically significant difference can be observed between the groups of low and high resilience in this dimension (*d* = 0.61; *p* = 0.05).

## Discussion

The way every patient copes with the disease and his/her ability for resilience, as well as his/her well-being levels, can make a difference regarding adaptation and adjustment to the disease. [44] The purpose of this research project was precisely to identify the most common coping strategies in patients with cancer, in order to find out if there is any relation between the type of coping and resilience, and if both of them are also related to a better quality of life in patients. The present study extends previous research by providing different groups of resilience levels, which allows to distinguish in what way different levels of resilience are related to coping and quality of life. This information might be significant for mental health professionals in order to support and advise their decision making process.

Results showed that most of the adaptive coping strategies were positively related to resilience, especially the strategies of “positive refocusing” and “positive revaluation”. López, Cantero and Marín [45] agree that the “positive thinking” strategy is one of the most commonly used by patients with cancer and they describe it as a variable that predicts better health benefits for the short and long term.

These results corroborate previous research projects that stated that there is a positive relation between the way of coping with the disease in an adaptive way and resilience. Smith et al [28] noted that resilience moderated the influence that coping with the disease had over the negative symptomatology in patients with cancer (anxiety, depression, stress. . .), thereby favouring the process of adapting to the disease. [46]

Results show that resilient people most commonly tend to cope with the disease in an adaptive way, which in the same time relates to favourable outcomes in terms of quality of life. These results match the ones obtained by Guil et al [47], who evaluated the relation between resilience, optimism and psychological well-being in women that had survived breast cancer. They found out that the level of resilience and well-being in participants was higher than the levels provided by the average values of the scales that were used to measure them. The fact of having experienced traumatic events could help people develop their capacity for personal growth and development, being able to bring meaning to that event, in the absence of denial of the experience. [3]

On the other hand, it has been noted that certain socio-demographic variables are related to the variables of coping and resilience. Older people used adaptive coping strategies to a lesser extent. [48] There are some studies that counter the negative effect of age over the way of coping with the disease. Similarly, results in this study show that resilience is lower in elderly; however, perception of their health increases as they get older. Although the physical decline is greater in the case of the elderly, results show that elderly people affected by cancer maintain an adequate emotional and cognitive balance, which could lead to a better perception of quality of life. [49,50] In fact, optimism, the search of social support and the use of certain coping strategies play a significant role in predicting a good emotional, cognitive and physical functioning. [51,52]

The findings of this research project suggest that coping with the disease in an adaptive way, as well as a high level of resilience, are related to a better adjustment to the oncological disease, in terms of a better quality of life. [53] Philip, Merluzzi, Zhang and Heitzmann [54] argue that the perception of a self-efficient way of coping with the disease (referring to an adaptive coping: appropriate affective regulation, stress management, active attitude. . .) can be important when adapting to the disease and it can also be a possible objective of the intervention. [19]

According to the results, resilience is positively and significantly related to the mental component of quality of life. In this regard, Liu, Zhang, Jiang and Wu [55] noted that resilience was partially involved in the relation between post-traumatic stress symptoms and the social support strategy in the case of people affected by cancer, thus favouring a greater psychological well-being. Likewise, Eicher et al [44] observed that a high level of resilience helps to improve the psychological well-being and quality of life in people with cancer. Indeed, they indicated that resilience is related to both mental health and physical health.

However, results in this study show that the physical component of quality of life is also related to resilience, but in a negative way. Patients undergoing oncological treatment could be suffering from numerous physical severe symptoms that directly influence their physical condition, such as bodily pain, fatigue, sleep difficulties, gastrointestinal or endocrine disorders, among others. [56] This relation could be derived from the influence of bodily pain, which is also negatively and significantly associated with resilience. A possible explanation could be that people who suffer from high levels of bodily pain, and consequently, worse physical quality of life, could be adopting a more resilient behaviour, in response to that distress. However, this result could be incidental as, conversely, many studies have found a positive association between resilience and physical quality of life. For example, Harms et al [57], found a link between resilience as a protective factor against the negative impact of adversity for certain components of physical quality of life, such as bodily pain. Similarly, Ristevska-Dimitrovska, Filov, Rajchanovska, Stefanovski and Dejanova [58] confirmed that all functional scales of quality of life (included the physical) were positively linked to resilience. Nevertheless, it was noteworthy that the symptoms severity, such as pain (among others), presented a negative relation with resilience. On the other hand, Walsh et al [59] conducted a mediation model in people with prostate cancer survivors and found that resilience had an important indirect impact on quality of life through physical posttraumatic growth (PPTG).

Finally, a number of restrictions have been found in this study. Firstly, the sample presents a great variability as it was composed with patients with different types of tumour or cancer diagnosis. This lack of homogeneity could compromise generalization of the results to every cancer populations. Additionally, findings may be limited to be generalized to other stages of the disease (later survivorship, palliative care…). For instance, results could be different if scores between patients in early stages and those who are suffering from a metastatic disease were analysed. It would be desirable to try to increase the sample homogeneity in further studies regarding these clinical variables.

Secondly, it would be also advisable to try to increase sample size in order to explore if statistical significance would increase. It would be interesting to analyse deeply if effect sizes would be greater when considering results by different resilience groups. Thirdly, methods for collecting sample could be improved; an effort should be done for homogenising modalities for answering the questionnaire (in paper or online) and it would desirable to conduct the participants’ selection by a randomized system.

There could be some bias because patients may have attended psychotherapy sessions, and that could affect the scores obtained in the variables that have been analysed cross-sectionally. Participants have received psychological support through AECC and therefore, the assessment of the disease and the way of coping with it could have been modified. Due to this fact, in the case of future research projects, it would be advisable to consider that participants may have received -or not- psychotherapy before completing the questionnaire. Finally, the cross-sectional design of the study is a limitation itself. A longitudinal study with at least two periods of evaluation would be suitable in order to explore predictive effects of resilience and coping strategies on cancer patients´ quality of life.

## Conclusion

By means of this study, the relation between resilience and the way of coping with the disease has been discussed, regarding the quality of life of people affected by an oncological disease. The resulting data does not allow to prove that variables have a predictive effect. Nevertheless, it has been proved that there is a substantial and positive association between resilience and coping with the disease in an adaptive way. In the same way, results show that both variables are likewise related to the quality of life of people with cancer. Thus, this study provides information about how different groups of resilience levels are related with coping and quality of life in people with cancer. It could be an interesting and useful information for psychologists in the oncological area who have to take decisions in the clinical context. The findings obtained in this study allow to know the clinical relevance of the results, beyond the fact that if has been a statistical significance or not, as it permits considering the changes taking into account the particular cases, in this case, the results in the different groups of resilience. Finally, a practical consequence of this evidence would involve trying to modify the way in which people affected by cancer cope with it, as well as increasing the level of resilient resources, so that these people can adapt themselves better to their disease process.
